# Supplementary material for: Gene drive designs for efficient and localisable population suppression using Y-linked editors
Source: PLoS Genet. 2022 Dec 27;18(12):e1010550. doi: 10.1371/journal.pgen.1010550 (PMC9829173; doi:10.1371/journal.pgen.1010550)
Supplement: S1 File — (DOCX) [file pgen.1010550.s001.docx]

# Supplementary information: the model and PAM site analysis

## Model organism and ecology

The model considers an infinite population with discrete non-overlapping generations, separate sexes and male heterogamety. Mating is random, and all females mate (i.e., males are not limiting). There are two life stages, juveniles and adults, and density-dependent and -independent mortality occur at the juvenile stage, with the chance of surviving to adulthood being equal to Θ * α / (α + N_h_[t]), where Θ represents non-specific density-independent mortality, α the strength of density-dependent mortality, and N_h_[t] the total number of juveniles at that time point. A given intrinsic rate of increase of the population (*R_m_*) is used to calculate the number of eggs produced per female. All population sizes are reported relative to the initial pre-release population size, and therefore *R_m_* is the only demographic parameter affecting the results – if this is kept constant, changes in Θ and α have no effect on the dynamics. Transgenic releases are assumed to be performed as releases of adult males that have equal mating success as wild males.

## Molecular implementation

While the proposed strategy can be implemented in a number of ways, the specific genetic instantiation we model consists of four different genetic elements (Fig. 1 in main text). We assume the YLE to be constructed using a CRISPR/Cas type RNA-guided endonuclease system made up of a nuclease component, i.e. Cas9, and a guide RNA (gRNA1) component that allows editing of the target gene. Further, we assume the autosomal element consists of an X-shredder nuclease and a second guide RNA (gRNA2) that allows the autosomal construct to home in the presence of the YLE. All components can independently become defective due to mutation.

In total, the model considers four different loci: a locus on the Y chromosome where the YLE would be inserted, an autosomal locus where the other construct would be inserted, a locus on the X-chromosome that is the target of editing, and another X-linked site that is the target of X-shredding. The last one may consist of an array of tandem repeats, but for simplicity we model it as a single locus. Given that each of the components of the insertions can acquire loss-of-function mutations, and each of the target sites can acquire resistant mutations, there are 5 different Y chromosome variants, 6 autosomal variants and 6 X chromosome variants (Fig A). All biologically permitted combinations of chromosomes lead to 1071 different genotypes being considered in the model.

**Y chromosomes**


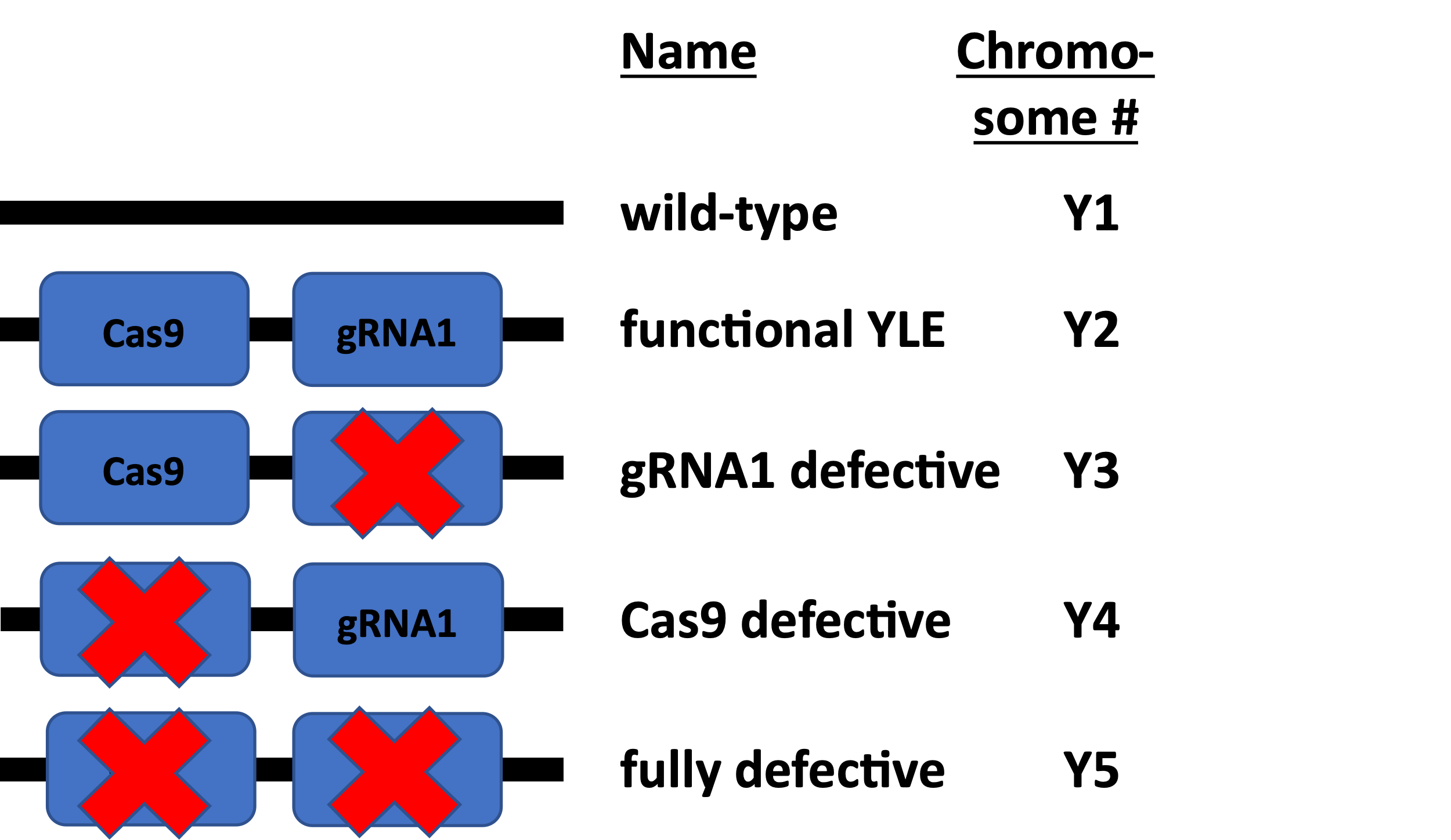


**X chromosomes**


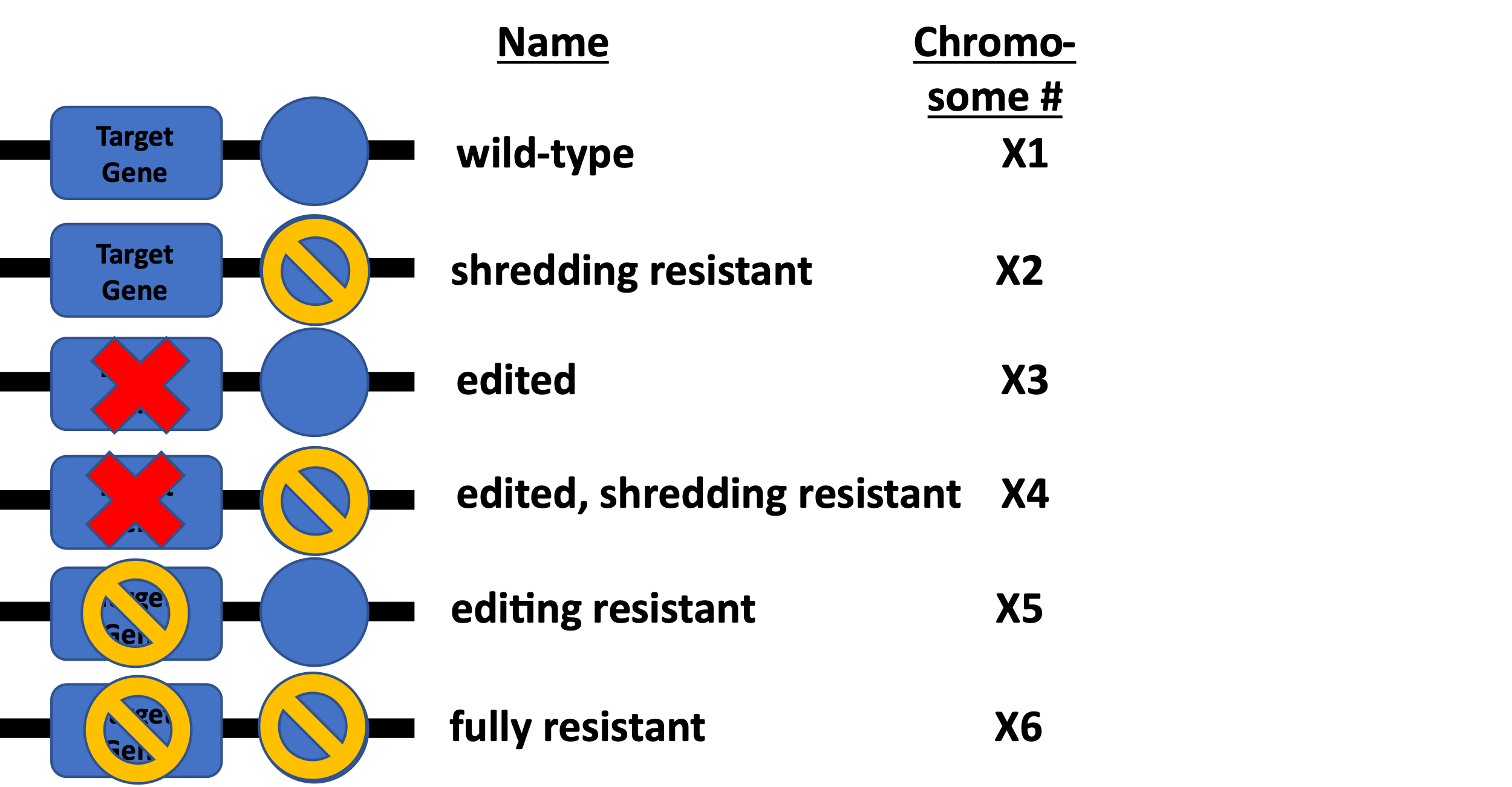


**Autosomes**


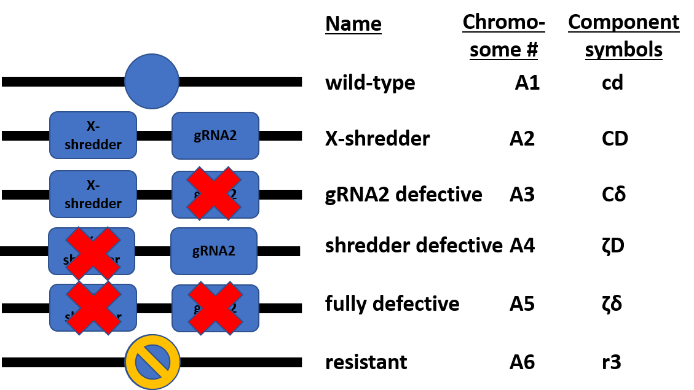


**Fig A** Overview of the different chromosome variants in the model

## Editing

Editing is assumed to occur in the germline of males carrying a functional YLE and an editable wild-type target gene. The frequency with which editing effectively occurs is described by the parameter *e_e_* (efficiency of editing). While the intention of the editing is to produce a non-functional or dominant negative allele, occasionally, with probability *e_r1_*, a functional allele that is equally fit as the wildtype and is resistant to further editing is produced (Fig B). The resulting editing-resistant variant is assumed to have equal (i.e., unaffected) fitness as the wild-type.


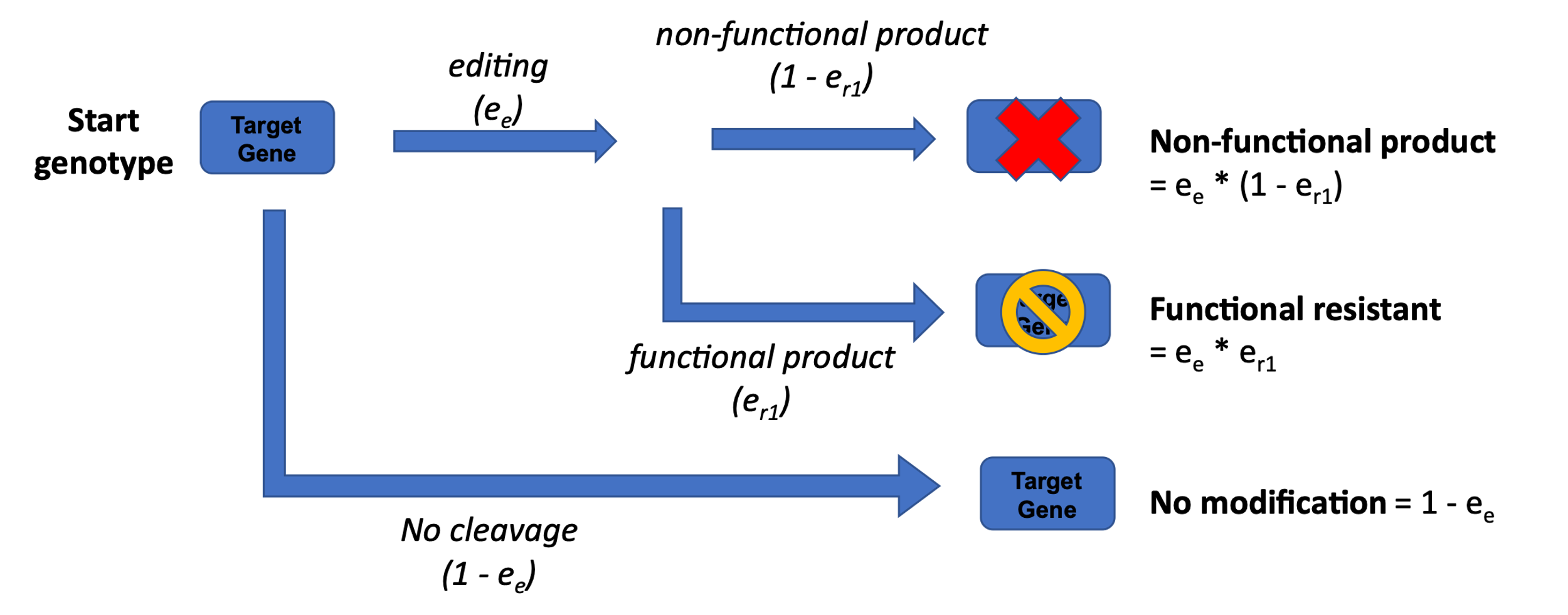


**Fig B:** Schematic representation of the editing process.

## Homing

Homing is assumed to occur when a functional Cas9, a functional gRNA2 and a wild-type autosome occur in the same individual. The homing process begins with cleavage of the wild-type allele, which occurs with probability *e_h_*. This is then repaired by end joining, creating a homing-resistant allele (probability *e_r3_*), or by homology-directed repair (probability 1- *e_r3_*). When there is homology-directed repair, the gRNA2 and the X-shredder (if it exists) may each acquire loss-of-function mutations during the homing process with probability *m_1_* (Fig C).


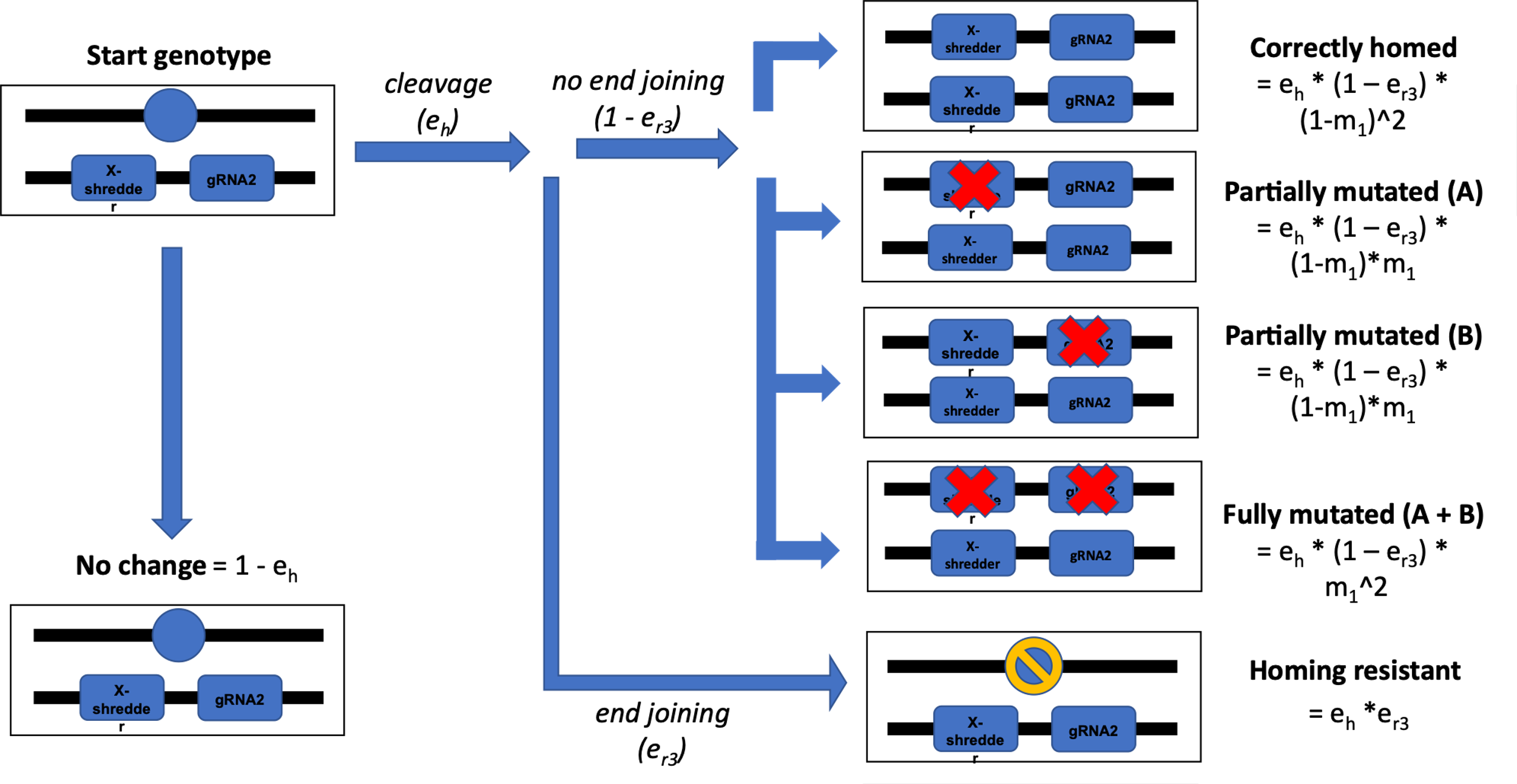


**Fig C.** Schematic representation of the homing process.

## Shredding

Shredding is assumed to occur during sperm production and distorts the frequency with which sex chromosomes are passed on. Parameter *e_s_* (efficiency of shredding) describes how frequently X chromosomes are shredded in X-shredder homozygotes, with *h_e2_* being its dominance coefficient for X-shredder heterozygotes. We assume throughout that the shredding rate is not dosage dependent (*h_e2_*=1). Shredded X-chromosomes are repaired and resistant to further shredding with probability *e_r2_*.

## Recombination

Recombination may occur, with probability *r*, between the X-linked targes of editing and shredding. It only has an effect in females that are double heterozygotes.

## Mutation

All transgenic elements become defective through loss-of-function mutations during normal DNA replication with probability *m_2_*.

## Selection

The fitness of wildtype genotypes is standardised to 1. For simplicity, all types of selection via imposed fitness costs are assumed to occur at the same time, via differential mortality at the adult stage (i.e., after density dependent larval mortality). Many different factors can reduce fitness. Parameter *s_f_* describes the fitness cost for females homozygous for the edited target gene, *h_f_* the corresponding dominance factor of that fitness cost, and *s_m_* is the fitness cost for males carrying an edited target gene. Fitness costs of the different transgenic elements are broken down into different categories to allow for partially defective variants. Parameter *s_a_* represents the expression cost of the Cas9 protein, *s_b_* the expression cost of a gRNA, and *s_c_* the expression cost of the X-shredder protein. Further, we suppose there is an additional activity associated cost when Cas9 is in the presence of a gRNA (*s_d_*) and an activity cost of the X-shredder (*s_e_*), which has dominance coefficient (*h_e_*) in heterozygous individuals. We assume throughout that the activity cost of X-shredding is not dosage dependent (*h_e_*=1).

## The order of events

The order of events is as shown in Fig D. All plots in the paper show the state of the population as censused at the 9th stage, showing somatic genotypes.


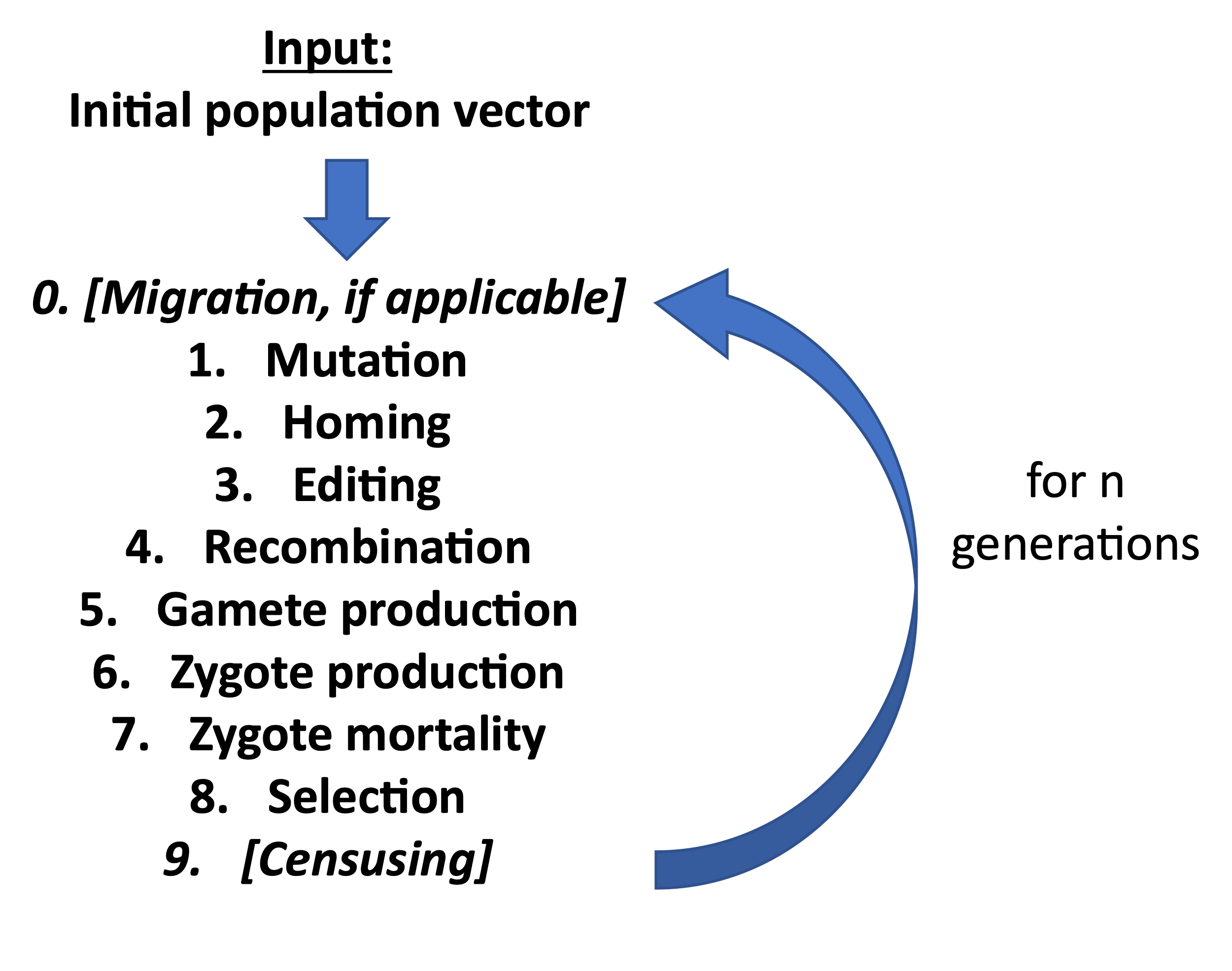


**Fig D.** The order in which different life cycle processes were modelled

## Calculating reproductive load

The reproductive load imposed by the constructs in a particular generation is defined as the proportionate reduction in the reproductive capacity of the population, and combines the effects on sex ratio and female fitness (following the assumption that the reproductive output of the population is not male limited), but is independent of ecological assumptions about the form and magnitude of density-dependent reproduction and survival. Load in a particular generation is calculated as:

$$\mathcal{L}=1-2f\overline{w_{f}}$$

where *f* is the proportion of zygotes that are female and $\overline{w_{f}}$ is the mean fitness of females:

$$\overline{w_{f}}=\sum_{i} p_{i}w_{i}$$

Here *p_i_* is the proportion of females that have genotype *i*, *w_i_* is their fitness (relative to wildtypes), and the sum is over all possible genotypes.

## Baseline parameter values

A list of parameters in the model and their baseline values for the idealised case and the sensitivity analyses in the main paper are shown in Table A.

| **Table A: List of parameters and their baseline values.**   \| Parameter  name \| Description \| \| \| Ideal baseline  value  (Fig 2 a + b) \| Including loss-of-function mutations (Fig 2 c + d) \| Including target site resistance (Fig 2 e - g) \| Sensitivity scan baseline value  (Fig 3 - 5) \| \| --- \| --- \| --- \| --- \| --- \| --- \| --- \| --- \| \|  \| \|  \| **Molecular efficiencies** \| \| \| \| \| \| *e_e_* \| efficiency of editing an X-linked target gene \| \| \| 0.95 \| 0.95 \| 0.95 \| 0.95 \| \| *e_s_* \| efficiency of X-shredding \| \| \| 0.90 \| 0.90 \| 0.90 \| 0.90 \| \| *e_h_* \| efficiency of cleaving the autosomal homing site \| \| \| 0.95 \| 0.95 \| 0.95 \| 0.95 \| \|  \| \|  \| **Fitness effects** \| \| \| \| \| \| *s_f_* \| selection coefficient against homozygous females carrying the edited X-linked gene \| \| \| 1 \| 1 \| 1 \| 1 \| \| *h_f_* \| dominance coefficient for heterozygous females carrying only one edited allele \| \| \| 1 \| 1 \| 1 \| 1 \| \| *s_a_* \| fitness cost of Cas9 expression \| \| \| 0 \| 0 \| 0 \| 0 \| \| *s_b_* \| fitness cost gRNA1 / gRNA2 expression \| \| \| 0 \| 0 \| 0 \| 0 \| \| *s_c_* \| fitness cost of X-shredder expression \| \| \| 0 \| 0 \| 0 \| 0 \| \| *s_d_* \| fitness cost of Cas9-gRNA complex activity \| \| \| 0 \| 0 \| 0 \| 0.01 \| \| *s_e_* \| fitness cost of X-shredder activity \| \| \| 0 \| 0 \| 0 \| 0.01 \| \| *s_m_* \| selection coefficient against males carrying an edited target gene \| \| \| 0 \| 0 \| 0 \| 0 \| \|  \| \|  \| **Loss-of-function mutation rates** \| \| \| \| \| \| *m_1_* \| mutation rate during homing \| \| \| 0 \| 1e-3 \| 1e-3 \| 1e-3 \| \| *m_2_* \| background mutation rate \| \| \| 0 \| 1e-6 \| 1e-6 \| 1e-6 \| \|  \| \|  \| **Probabilities of resistance arising** \| \| \| \| \| \| *e_r1_* \| probability the edited allele is functional and resistant to further editing \| \| \| 0 \| 0 \| 0, 0.05 \| 0 \| \| *e_r2_* \| probability a shredded X-chromosome is repaired and resistant to further shredding \| \| \| 0 \| 0 \| 0, 0.05 \| 0 \| \| *e_r3_* \| probability the cleaved target of homing is repaired by end-joining \| \| \| 0 \| 0 \| 0, 0.05 \| 0.05, 0.6 \| \|  \| \|  \| **Miscellaneous** \| \| \| \| \| \| *r* \| recombination rate of X-linked targets of editing and shredding \| \| \| 0.5 \| 0.5 \| 0.5 \| 0.5 \| \| *R_m_* \| intrinsic rate of population growth \| \| \| 6 \| 6 \| 6 \| 6 \| |
| --- | --- | --- | --- | --- | --- | --- | --- | --- | --- | --- | --- | --- | --- | --- | --- | --- | --- | --- | --- | --- | --- | --- | --- | --- | --- | --- | --- | --- | --- | --- | --- | --- | --- | --- | --- | --- | --- | --- | --- | --- | --- | --- | --- | --- | --- | --- | --- | --- | --- | --- | --- | --- | --- | --- | --- | --- | --- | --- | --- | --- | --- | --- | --- | --- | --- | --- | --- | --- | --- | --- | --- | --- | --- | --- | --- | --- | --- | --- | --- | --- | --- | --- | --- | --- | --- | --- | --- | --- | --- | --- | --- | --- | --- | --- | --- | --- | --- | --- | --- | --- | --- | --- | --- | --- | --- | --- | --- | --- | --- | --- | --- | --- | --- | --- | --- | --- | --- | --- | --- | --- | --- | --- | --- | --- | --- | --- | --- | --- | --- | --- | --- | --- | --- | --- | --- | --- | --- | --- | --- | --- | --- | --- | --- | --- | --- | --- | --- | --- | --- | --- | --- | --- | --- | --- | --- | --- | --- | --- | --- | --- | --- | --- | --- | --- | --- | --- | --- | --- | --- | --- | --- | --- | --- | --- | --- | --- | --- | --- | --- | --- | --- | --- | --- | --- | --- | --- | --- | --- | --- | --- | --- | --- |

PAM site analysis

Our analyses used population genomic sequence data from 1138 individuals from 15 populations, including *An. gambiae* ss, *An. coluzzii*, and populations of uncertain assignment, since gene flow is known to occur between them. The populations and sample sizes are as follows: *An. gambiae* from Cameroon (594), Uganda (224), Burkina Faso (184), Gabon (138), Guinea (80), Mayotte (48), Ghana (24), Bioko (18); *An coluzzii* from Angola (156), Burkina Faso (150), Cote d’Ivoire (142), Ghana (110); and populations of uncertain assignment from Guinea-Bissau (182), The Gambia (130) and Kenya (96) where n is the number of sequences and n/2 is the number of individuals sampled.

The frequencies of potential PAM sites (i.e., GG and CC dinucleotides) were calculated for each population, and sites with more than 5% missing data within any population were removed, leaving 13,462,450 polymorphic PAMs. For each population pair we counted the number of PAM sites which were present in one population at >36% frequency and in the other at <13%, representing the level of differentiation required at the homing target site in low EJR species to limit impact to the first population, then reversed the assignment of populations (as if the second was the target), and then averaged the two counts. The analysis was then repeated using the thresholds appropriate for high EJR species (>88% in one population and <33% in the other). F_ST_ values were obtained from *Anopheles gambiae* 1000 Genomes Consortium [1] (2020, Genome Res. 30:1533-46, Supplemental Fig S5). The sample site map was generated using the cartopy python package.

References

1. The Anopheles gambiae 1000 Genomes Consortium. Genome variation and population structure among 1142 mosquitoes of the African malaria vector species Anopheles gambiae and Anopheles coluzzii. Genome Res. 2020;30(10):1533-46. doi: 10.1101/gr.262790.120. PubMed PMID: WOS:000577121300013.
