# Supplementary material for: Gene drive designs for efficient and localisable population suppression using Y-linked editors
Source: PLoS Genet. 2022 Dec 27;18(12):e1010550. doi: 10.1371/journal.pgen.1010550 (PMC9829173; doi:10.1371/journal.pgen.1010550)
Supplement: S1 Fig — Timecourse of gene and population dynamics when only one construct is released, either the YLE (a) or the ASD (b) for the idealised case of no mutation, no resistance, and no unintended fitness costs. (DOCX) [file pgen.1010550.s002.docx]

**S1 Fig.** Timecourse of gene and population dynamics when only one construct is released, either the YLE (a) or the ASD (b) for the idealised case of no mutation, no resistance, and no unintended fitness costs.
