# Supplementary material for: Gene drive designs for efficient and localisable population suppression using Y-linked editors
Source: PLoS Genet. 2022 Dec 27;18(12):e1010550. doi: 10.1371/journal.pgen.1010550 (PMC9829173; doi:10.1371/journal.pgen.1010550)
Supplement: S2 Fig — A YLE by itself can give good suppression with repeated 10% releases (a), but a single 10% release has little effect (b). A single 10% release of a YLE combined with a non-driving ASD can give good suppression (c), but a single release of 0.1% has little effect (d), whereas a 0.1% release of the double drive design considered in this paper can give good suppression (e). Note that the editing and shredding rates were set to maximum values (1) and released males are homozygous for the ASD in Burt & Deredec (2018), whereas they are slightly smaller in this publication (0.95 and 0.9, respectively) and the released males are heterozygous. (DOCX) [file pgen.1010550.s003.docx]

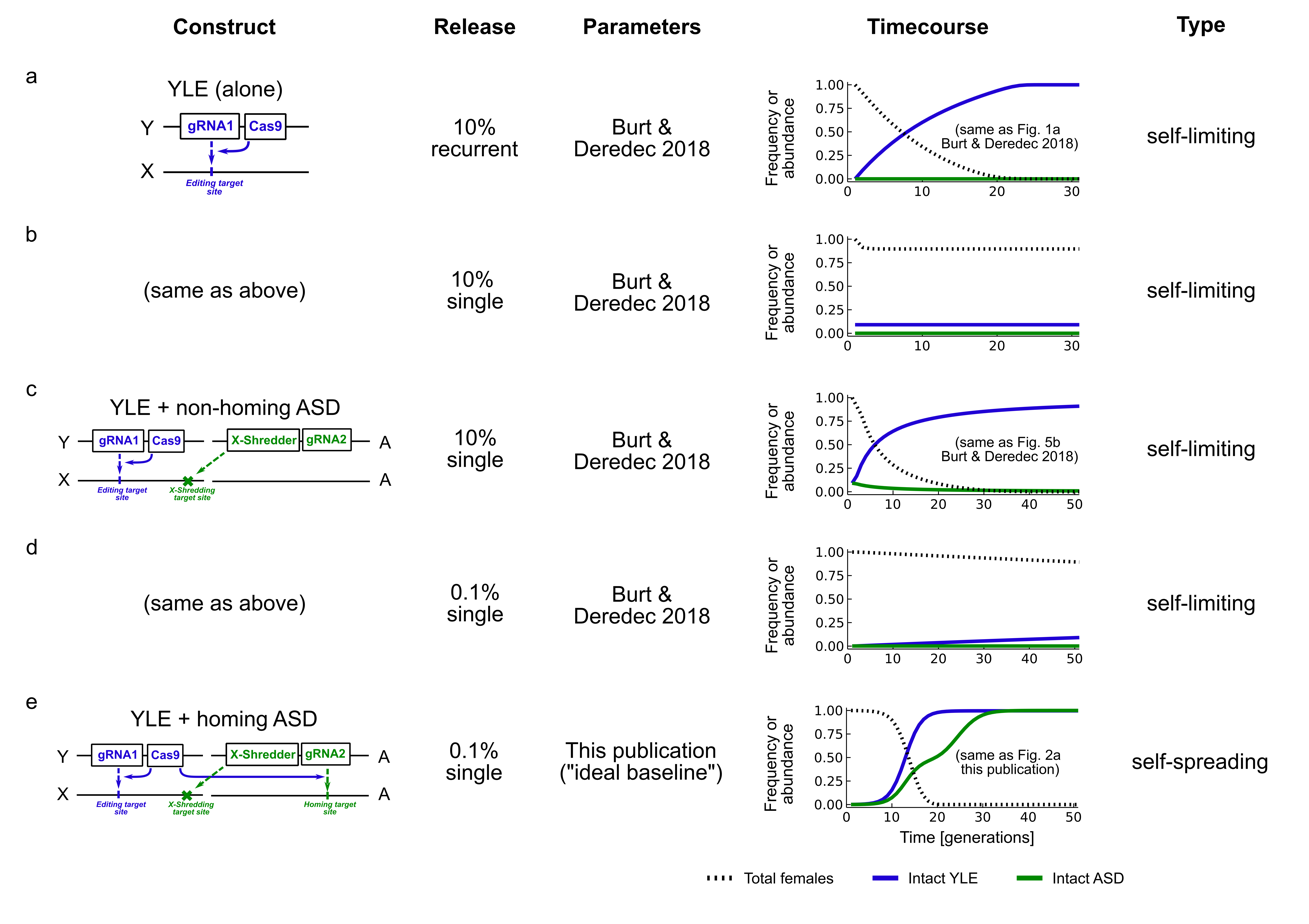


**S2 Fig.** Comparison of alternative population suppression designs using YLEs. A YLE by itself can give good suppression with repeated 10% releases (a), but a single 10% release has little effect (b). A single 10% release of a YLE combined with a non-driving ASD can give good suppression (c), but a single release of 0.1% has little effect (d), whereas a 0.1% release of the double drive design considered in this paper can give good suppression (e). Note that the editing and shredding rates were set to maximum values (1) and released males are homozygous for the ASD in Burt & Deredec (2018), whereas they are slightly smaller in this publication (0.95 and 0.9, respectively) and the released males are heterozygous.
