# Supplementary material for: Gene drive designs for efficient and localisable population suppression using Y-linked editors
Source: PLoS Genet. 2022 Dec 27;18(12):e1010550. doi: 10.1371/journal.pgen.1010550 (PMC9829173; doi:10.1371/journal.pgen.1010550)
Supplement: S3 Fig — Each plot shows the minimum population size as a function of changes in the specified parameter assuming low EJR (5%; dotted lines) or high (60%; dashed lines) and intrinsic rate of increase of either 2 (orange), 6 (black) or 12 (purple). Note for (a) that the homing rate is equal to (cleavage rate) x (1-end joining rate); for (e) that the orange dotted line is below the y axis limit (1e-10); and for (m) that only 1 line is shown for each end-joining rate since the intrinsic rate of increase is the dependent variable on the x-axis. (DOCX) [file pgen.1010550.s004.docx]

| 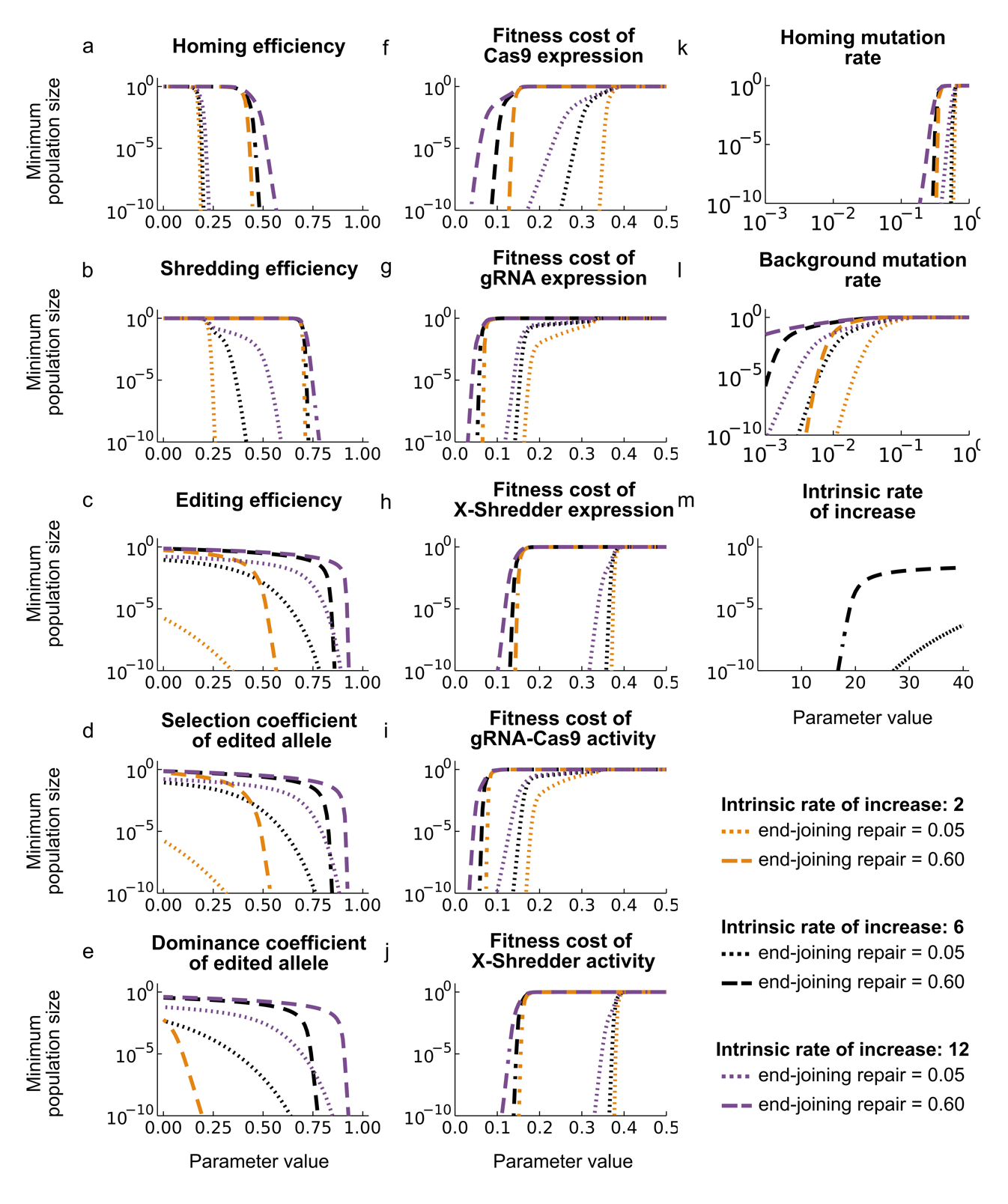 |
| --- |
| **S3 Fig.** Extended sensitivity analysis. Each plot shows the minimum population size as a function of changes in the specified parameter assuming low EJR (5%; dotted lines) or high (60%; dashed lines) and intrinsic rate of increase of either 2 (orange), 6 (black) or 12 (purple). Note for (a) that the homing rate is equal to (cleavage rate) x (1-end joining rate); for (e) that the orange dotted line is below the y axis limit (1e-10); and for (m) that only 1 line is shown for each end-joining rate since the intrinsic rate of increase is the dependent variable on the x-axis. |
