# Supplementary material for: Gene drive designs for efficient and localisable population suppression using Y-linked editors
Source: PLoS Genet. 2022 Dec 27;18(12):e1010550. doi: 10.1371/journal.pgen.1010550 (PMC9829173; doi:10.1371/journal.pgen.1010550)
Supplement: S4 Fig — (DOCX) [file pgen.1010550.s005.docx]

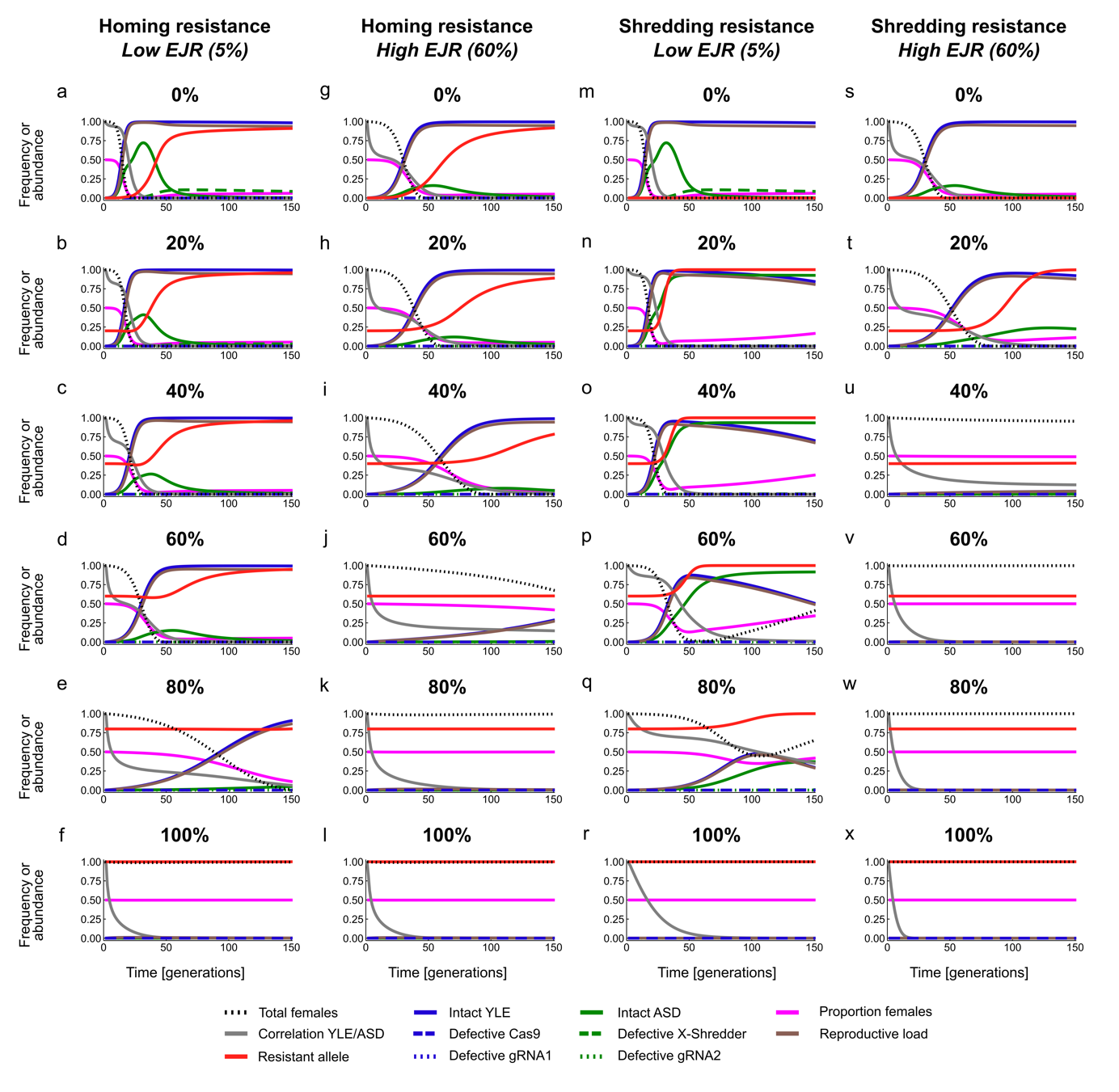


**Supplementary Figure SF-4.** Timecourses for gene and population dynamics with different initial frequencies (0, 20, 40, 60, 80 or 100%) of either homing or shredding resistance in low and high EJR species.
