# Supplementary material for: Gene drive designs for efficient and localisable population suppression using Y-linked editors
Source: PLoS Genet. 2022 Dec 27;18(12):e1010550. doi: 10.1371/journal.pgen.1010550 (PMC9829173; doi:10.1371/journal.pgen.1010550)
Supplement: Attachment — Submitted filename: v2 Responses to comments.docx [file pgen.1010550.s006.docx]

Responses to editor’s and referees’ comments

In general, both reviewers and myself are favorably disposed to the manuscript, and it can most likely be published with some revisions and additions (because of the likely need for data additions, I would classify this as a major revision). Please address all reviewer questions and comments in your response. I have a few additional comments.

If possible, it would be helpful to show **genetic load at the time of peak population** **reduction** to allow for easier cross-comparisons with other suppression drives. This is a more general property than reduction in population size, which is heavily dependent on the specific species, ecology, and model used.

*This is a good suggestion, and we have added a line for load to Figure 2, with corresponding description in the text of the results, and a description of how load is calculated in the methods.*

In the discussion, the authors state that, “This can be done by targeting key functional sites that are not able to change while maintaining function, and by targeting **multiple neighbouring** sites **in the same target gene** (Champer et al. 2018; Kyrou et al. 2018; Champer et al. 2020). By comparison, selection for resistance at the sites of homing and shredding is weaker, and higher rates of mutation to resistance can be tolerated.”
In fact, the **target sites in this gene need not be neighboring.** This is a requirement for efficient homing drives with multiple gRNAs, but not for the toxin component of gene drives, for which end-joining repair (and thus more widely spaced gRNAs) is fine (Champer et al, BMC Biology, 2020). Even **targeting different genes should be equally feasible in this situation.**

*Agreed, we have removed ‘neighbouring” and added “or even in different genes.”*

This later point may be important if there are only 4000-8000 differentiable targeting sites. In this case, most genes won’t even have a single site. It might be necessary to have multiple possible targets, though even **finding one haploinsufficient gene on the X-chromosome with a differentiable target might be very difficult.** Unless I misunderstand this, perhaps a bit more skepticism for this method could be incorporated into the discussion. of course, I suppose not every target needs to be differentiable (especially the X-shredder), but the X-linked and probably the ASD gRNA target should be.

*This appears to be a misunderstanding. We do not require a differentiated target site in the X-linked haplo-insufficient gene (which, if necessary, would indeed limit the applicability of sequence-based localisation). In the Results we state: “As noted above, 100% editing resistance can still lead to the spread of the constructs and some suppression, so choosing a site for editing that is differentiated between target and non-target populations will not provide effective restriction.” Rather, the analyses in Figure 5 show how restriction can be accomplished using differentiation either at the insertion site of the ASD (“homing resistance”) or at the target of X-shredding (“Shredding resistance). Either one by itself can do. We have added a sentence to the discussion to make this more clear: “Such localisation can be achieved by choosing the insertion (and homing) site of the ASD or its target site on the X chromosome (or both) to have a suitably differentiated sequence.”*

Another possible consideration is that the effect of mutations in the ASD component could perhaps be reduced if placed in a haplosufficient but essential female gene (much like the construct in Simoni et al, Nature Biotechnology, 2020). (The authors may have already intended this in which case ignore this comment). In this situation, if the construct loses function of its components (particularly the X-shredder) due to mutating, the allele would be less fit because it would be removed in females with combinations of ASD alleles. Normally, ASD alleles may not often be together because of the X-shredder component, which tends to keep the drive in male drive/wild-type heterozygotes.

*This is an interesting idea, and we have added the following sentence to the discussion: “In principle, it may be possible to increase the evolutionary stability of the ASD by inserting it into a female-essential gene (Simoni et al. 2020), as then mutants losing the X-shredder function would occur more often in females, and be selected against, though further modelling would be needed to confirm this idea, and our results suggest it may not be necessary.”*

Reviewer 2’s comment on sensitivity analysis is quite important to address, but it may be impractical to conduct a full sensitivity analysis on all possible combinations without bringing in some additional computational techniques and a large amount of additional data collection (due to the large number of parameters investigated - for an example see Sam Champer et al., PLOS Computational Biology, 2021). Perhaps the authors could use a combination of discussion together with a somewhat more limited set of new analysis or data to address this (focusing on the parameters deemed most likely to be important, most likely to be variable, and most likely to have interesting interactions with others).

*See our response to Reviewer 2 below.*

Reviewer's Responses to Questions

**Comments to the Authors:
Please note here if the review is uploaded as an attachment.**

Reviewer #1: In this manuscript, the authors describe and model a suppression gene drive that builds upon their previous work with Y-linked editors and autosomal sex distorters. They introduce a tweak by adding a gRNA that facilitates homing of the ASD element. The YLE is designed to create a dominant mutation on the X causing lethality or sterility in females, resulting in population suppression.
Although, as the authors point out, the molecular implementation might be difficult (expression from the Y), I really like the design. With its location on the Y, the main load inducing component in form of the editor is shielded from the negative effects it creates. When the YLE is present in an individual that also carries the shredder both elements get a boost in frequency. The YLE induces homing of the ASD and the ASD ensures that most of the offspring will inherit a Y and thus, the YLE.

*We thank the reviewer for their positive comments.*

The authors model population dynamics over a broad range of parameters, including LOF mutations of components, resistance at target sites, NHEJ events during homing, and fitness costs. They show that suppression works well for reasonable assumptions of these parameters. A single release with a low introduction percentage was used for all models. Thinking about a real world application, I would assume that multiple releases can be carried out, making suppression even stronger and working over a broader range of parameters as the modeling in the paper suggests.

*We expect the main effect of varying the size or number of releases to be on the time to impact rather than the level of eventual impact. This is because the construct can drive from arbitrarily low frequency, however the lower the introduction frequency, the longer it will take for it to spread through the entire population. We have added a sentence to the discussion: “All our simulations assume a single release of 0.1% of the original population size, and, as with other self-spreading gene drive systems (North et al. 2019; North et al. 2020), we expect the main effect of having larger or repeated releases would be to reduce the time to impact.”*

Finally, the authors touch on the subject of drive confinement based on target site diversity between target and non-target populations and give an estimate on how many such sites can be identified in Anopheles populations.

I think this clever combination of existing mechanisms is a great addition to the ever expanding drive system toolbox.

*Again, we than the reviewer for their positive comments.*

Comments:

The paper is based on previous work by some of the authors on YLE with non homing ASD. From looking at the old work, the biggest difference seems to be in the case of separate releases of the elements with a constitutive active ASD which did not work well without a homing ASD. A plot comparing the old and new systems for a simple set of parameters could help emphasize the advantages of the YLE with a homing ASD.

*The primary difference between the design analysed here and the previous one is that this one is self-spreading from a low frequency, whereas the previous one is self-limiting and therefore non-trivial release rates are required. We have added an extra figure in the SI to make this clear (Supplementary Figure SF-2). We have also added a sentence in the results “This ability to spread through and crash a population from very small releases is what distinguishes this double drive design from previously proposed YLE strategies, whether used alone or with a non-driving ASD (Supplementary Figure SF-2)“ and a sentence to the discussion: “The ability of the constructs to spread and have an impact from arbitrarily small release rates is the key distinguishing feature of our double drive design compared to the self-limiting augmented YLE designs in which the ASD does not have a gRNA and cannot home (Burt and Deredec 2018).”*

Page 4 Methods: The authors say that a number of different molecular constructions could be used. For readers trying to build such systems it might be helpful if the authors could sketch out these different designs (maybe in a supplemental figure?).

*We have expanded the section on this in the Discussion, so it now reads: “Some of the molecular options for the editor are outlined in Burt and Deredec (2018), including knocking out a haplo-insufficient gene, producing a dominant negative mutation, or using paternal deposition of the editor, and a proof-of-principle demonstration in D. melanogaster is provided by Fasulo et al. (2020). Instead of using a cleavage-based editor like Cas9 it may be possible to use a base editor or prime editor (Bosch et al. 2021; Marr and Potter 2021), and instead of targeting an X-linked locus, it would also be possible to target one on an autosome, but the impact would have to be female-specific; potential targets are rare but do exist (Navarro-Paya et al. 2020; Willis and Burt 2021).”*

Page 6: Is there an advantage in releasing the drive components in separate individuals? Does that help with the rearing of the strains? Why would one want to do that?

*There is not necessarily an advantage; we think this is interesting to show because it helps to better understand the processes underlying the dynamics. We have added a sentence: “To better understand the processes underlying these dynamics, we analysed the consequences of releasing the two constructs in different males (Fig. 2b).”*

Page 7: 1 in 1000 incorrectly homed alleles seems low. Is this number inferred from the cited publications?

*We are not aware of any good estimate of this quantity (and therefore not sure the basis on which this is thought to be ‘low’), and in any case it is likely to depend on the construct (e.g., how long and how repetitive it is – see the Simoni et al. paper cited). The key point is that the strategy seems to be robust to very high rates (ca. 50%; Fig. 4k).*

Page 9: What are the baseline values? The ones from page 7 or is everything else at 100% efficiency? Can the authors clarify?

*We now clarify that these are the values given in Supplemental Table S-1 and have expanded the table to explicitly state which parameter values were used in the different scenarios.*

Page 11: The authors perform a preliminary analysis to identify PAM sites that differ in frequency between target and non-target populations based on PAM sequence motif. There are additional restrictions that should be mentioned. In case of shredding, the PAM needs to be on the X (in multiple copies?). In the case of homing the PAM needs to be at a neutral site (not disrupting anything essential).

*We now include the sentences in the discussion: “We have not investigated whether these differentiated PAMs tend to be in coding or non-coding regions; if in the former, the ASD may need, for example, to be placed in an artificial intron (Nash et al. 2022) to reduce the fitness effects of gene disruption. We have also not investigated differentiation on the X chromosome, and it is not clear the extent to which a shredding strategy would need to target a repeated sequence.”*

Figure 6: (high EJR scenario, blue dots). Should that be gray dots?
Is it possible to highlight the immediately neighboring populations in the plot, since those might be most relevant when trying to prevent drive from spilling over?

*Yes, the caption has been corrected to now read gray. We do not think it would be useful to highlight neighbouring populations, as Anopheles mosquitoes may well disperse to distant populations based on assisted movement (e.g., on vehicles or on winds at high altitude); to only rely on differentiation with neighbouring populations would be risky.*

Page 14: The authors write...A particularly attractive feature of our design is the potential it offers for localization of the spread and impact of the releases based on preexisting sequence differences between target and non-target populations...
Wouldn't all (or most) sequence based drives have the potential for localization based on target sequence differences?

*The point is that some designs are much better at this than others. We have re-written a subsequent sentence to make this more clear: “Willis and Burt (2021) have previously shown that autosomal double drive designs may be able to work much better for population restriction than single construct designs, and with the design analysed here even smaller differences in allele frequency can be exploited, as seen in the relatively steep change in outcome based on small differences in allele frequencies (Fig. 5).”*

Reviewer #2: Geci et al. describe a double drive design that has a Y-linked editor (YLE) and an autosomal sex distorter (ASD), which help each other to 'drive'. YLE targets an essential gene on the X-chromromose (haplo-sufficient or haplo-insufficient) that cause lethality or sterility in the daughters. The editting takes place in the male germline, and does not rely on sex chromosome expression during meiosis, which is an advantage for some species. The ASD targets repeated sequences in the X chromosome (seperate from the YLE target) and shreds the chromosome during meiosis (which produces skewed sex ratio, driving the YLE). ASD can also home in the presence of the YLE in the germline, so YLE is 'driving' ASD. Neither contruct can 'drive' when the other is absent.

In order to investigate the spread of this double drive, Geci et al. develop a population genetic and population dynamic model (infinite population size) that is deterministic and panmictic (nonspatial) with nonoverlapping generations. These properties make it hard to extrapolate the results of the model to more realistic scenarios, however, it still make useful predictions that show this YLE-ASD double drive could suppress a population with low release rates and with no invasion threshold. They also show that the An. gambiae has thousands of suitable genomic sites that are differentiated even for weakly differentiated populations, which could restrict the spread of the drive to other non-target populations.

I would recommend Geci et al.'s work for publication with minor revision. I have three major comments: Firstly, I find the model description very short, I strongly suggest more detailed description of the model (e.g. in Willis and Burt, bioRxiv; and/or Burt and Deredec 2018).

*We are unsure about this comment, since the description given in the main text is about as detailed as that for Willis & Burt, and the SI gives as much information on the model as in the previous papers. However, the description in the SI was confusing, and we have amended it (see below).*

*The entire model and all files are freely available on GitHub. We have made the GitHub repository more accessible and added a readme and short introduction.*

Secondly, even though Geci et al. carry out analyses for multiple scenarios (loss of function, evolution of resistance through nonhomologous end joining, fitness costs etc.), they do it with one at a time approach (local sensitivity analysis). Since they do not vary multiple factors at the same time, it is not possible to understand how the interaction among various parameters effect the results particularly if they interact nonlinearly. Are there truly no nonlinear interactions between any of the parameters presented? Either that should be convincingly presented, or a global sensitivity analysis should be carried out where parameters of interest are varied simultaneously.

*We are certainly not claiming there are no non-linear interactions between the different parameters. However, we are not convinced that a global sensitivity analysis would be a significant contribution for the reader – we believe the main point is to identify which of the parameters are the most important, so, for example, someone deciding whether to try and build one of these in a species can judge whether the biology of the species is conducive to it. After that, it is up to the skill (and luck) of the engineer how well the different parameters can be optimised, and, at the end of the day, they will want to assess the specific combination of parameter values that they have been able to achieve to decide whether they should go to the next step. This assessment would be better done using our simulation model than using a statistical model generated using randomly chosen parameter values (though an even better approach would be plug the parameter values into a bespoke model tailored to the target species and environment, which is what would happen in practice).*

*For these reasons, our sensitivity analyses changed one parameter at a time. The one exception was we showed the results for 2 different values of the end joining rate, as this parameter may be something that the investigator may have little control over. We have extended this logic to now also consider 3 different values of the maximum rate of population increase (Rm), as that is the other factor that will be beyond the control of the molecular engineer. However, this plot is now a bit busy, and so we have put it in the SI, for the interested reader, and added the following to the results: “All these plots are for our baseline R_m_ value of 6; additional sensitivity plots for alternative values of 2 and 12 are shown in Supplementary Figure SF-3.”*

Lastly, given the assumptions of the model, more discussion should be done on the caveats of their model (panmictic, infinite population size, etc).

*We have added to the discussion: “we have not considered the effects of finite population, overlapping generations, spatial or temporal heterogeneity, and other factors that one would want to include in a model tailored to predict more precisely the dynamics and impacts of a release in a specific potential use case.”*

I also have a few minor comments below, which I hope the authors would find useful.

p.5. Could you provide more detail on the model code?

*We have provided a link to GitHub and added a read me introduction*

p.6. fig 2. I would recommend keeping the x axis range of a and b same as the others [0,150] for quick and easy visual comparison for all.

*We have adapted the figure.*

-Specify correlation in the legend as well (between YLE and ASD)

*We clarified this in the legend.*

-It might be useful to include the sex ratio skew in the graphs as well? I wonder why the females not increasing after defective x-shredder increases around generation 100 in 2c? Probably hard to see because of the reduced population size, in that case sex ratio plotted on the same graph would be also be useful...

*We have added the sex ratio (as proportion of females) to the figures.*

-Subtitle in c and d would be useful (e.g. “X-shredder” in c) like in other subfigures.

*Since we are not showing different scenarios in these subplots, we think it would confuse the reader to add subtitles to these plots. In c we are not treating the X-shredder any differently than any other component, it is just the one that responds differently.*

p.7. I am a bit confused about loss of function in X-shredder: “Despite the loss of the X-shredder, the population is still very substantially suppressed, because the YLE is still very frequent: the X-shredder functions primarily as a booster of the YLE, not as a source of reproductive load itself.” (also on Fig. 2c). Since YLE doesn't work by itself, later loss of X-shredder doesn't affect because by then YLE has reached really high proportions? (what is the critical high frequency, below which it will fail?) Could you explain a bit more? (sort of similar to Burt and Deredec 2018 where release ratios are higher with continuous releases for many generations).

*The YLE does not increase in frequency by itself, but it does work by itself to produce deleterious edits and a reproductive load.*

*We have reworded this passage to hopefully be clearer: “Nevertheless the population is still very substantially suppressed because, before being lost, the X-shredder boosts the YLE to a very high frequency, and the YLE is the main source of the reproductive load. While the X-shredder does contribute directly to the load, its main function is indirect, via boosting the frequency of the YLE.”*

p.10. Fig. 4. I would recommmend separeting the subfigures to different rows of relevant figures for easy visualization/interpretation (a-c), (d,e), (f-j), (k,l), and m.

*Following this suggestion would lead to an unequal number of plots per row (3,2,5,1) which would be visually inefficient. However, we did try to follow up on this suggestion by rearranging all plots into one column that have the same x axis limits.*

-Why are figures 4c and 4d (almost?) identical? Could you explain?

*They are not exactly identical, just very similar. And this is the case because, practically speaking, the effect of “having X% less edits being made by the YLE” (which is what lower editing efficiency would mean) is very similar to “same number of edits are being made but they cause X% lower fitness effect” (which is what a lower selection coefficient of edited would mean).*

p.12. Fig .6. should “sl” be “s.l/”?

*Corrected*

blue dots should be light gray?

*Corrected*

Supp. Fig. S5 in the reference?

*The cited paper is a long paper, and given the data is tucked away in the paper’s SI, we thought it useful to point the reader to where exactly in the paper the data can be found.*

The scale of the inset does not allow all 15 to be seen.

*Two of the points were hidden behind other ones. We have now modified the figure to show the different subspecies, and this then makes the overlapping of points clear.*

p.20. Supplementary Figure SF-2: third column title should be “low ejr (5%)”
fig. Caption “with” different initial
frequencies?

*Corrected*

p.21. Supplementary information, the model: could you give more details on the model also the parameters used on mortality, currently only intrinsic rate of increase of the population is given in the supp. Table. Is theta density-dependent or independent?

*Theta is meant to be “density-independent” mortality, and this has been corrected.*

*More importantly, we have added a sentence to make clear that the only demographic parameter affecting the dynamics is Rm: “All population sizes are reported relative to the initial pre-release population size, and therefore R_m_ is the only demographic parameter affecting the results – if this is kept constant, changes in Θ and α have no effect on the dynamics.”*

You mention “7 X chromosome variants (Supplementary Figure SF-3)” but the figure has 6 and 1071 can be obtained by 6 X chromosome variants.

*Corrected.*

p. 25. Supplemental Table S-1: The range of values tested for sensitity analysis as well, rather than only the baseline values would be useful. (also as I have mentioned earlier, SA should be repeated with multiple parameters varied simultaneouly.)

*As indicated above, we do not think it useful to vary the parameters simultaneously, and so there is little point in showing the range in the table – the range can be easily seen in the appropriate panel of Fig. 4 and Fig SF-3.*
